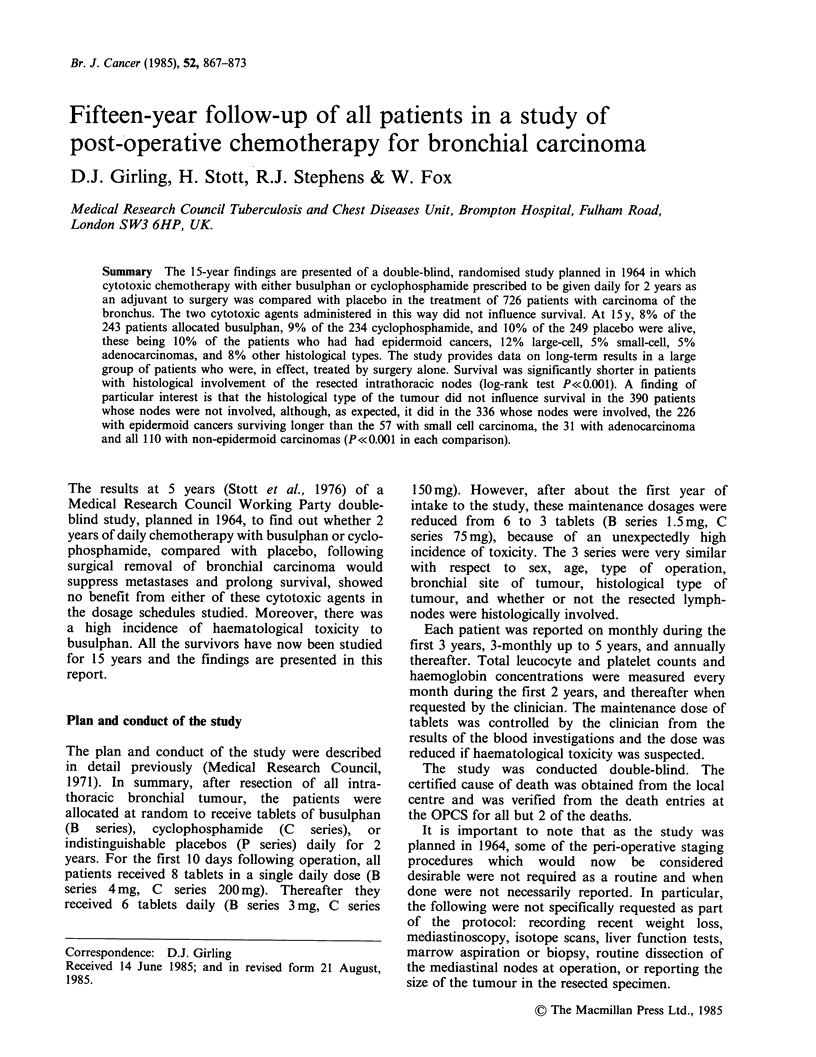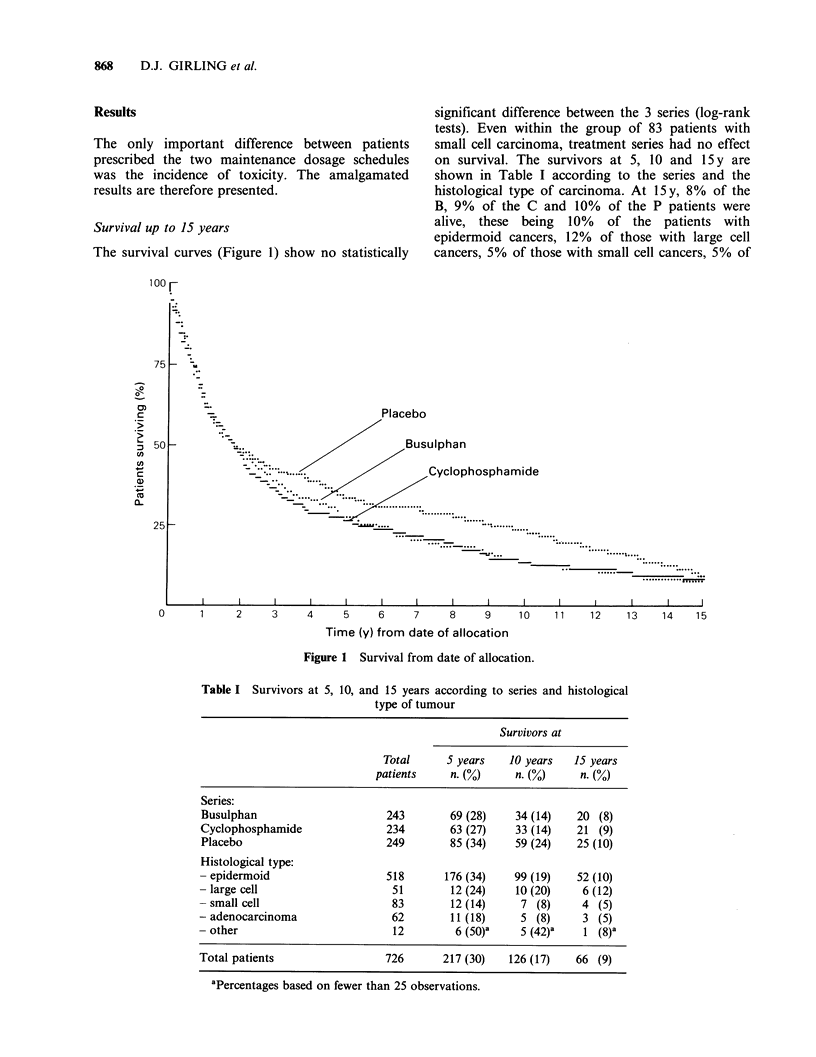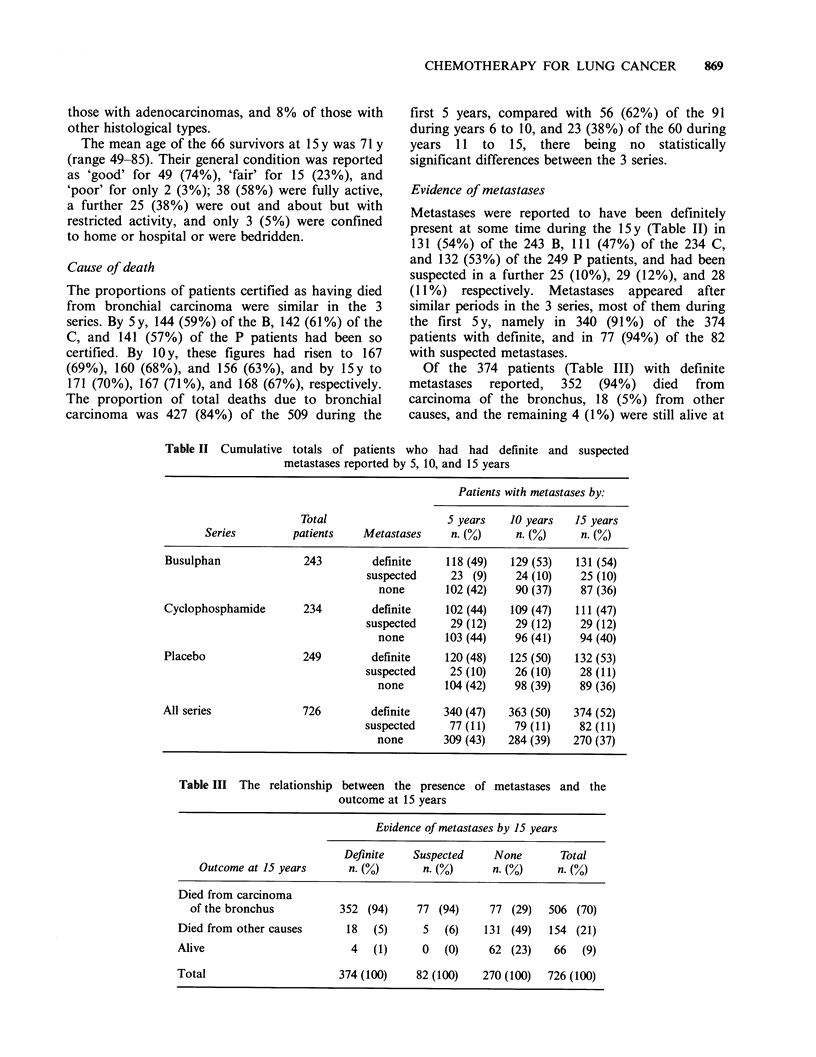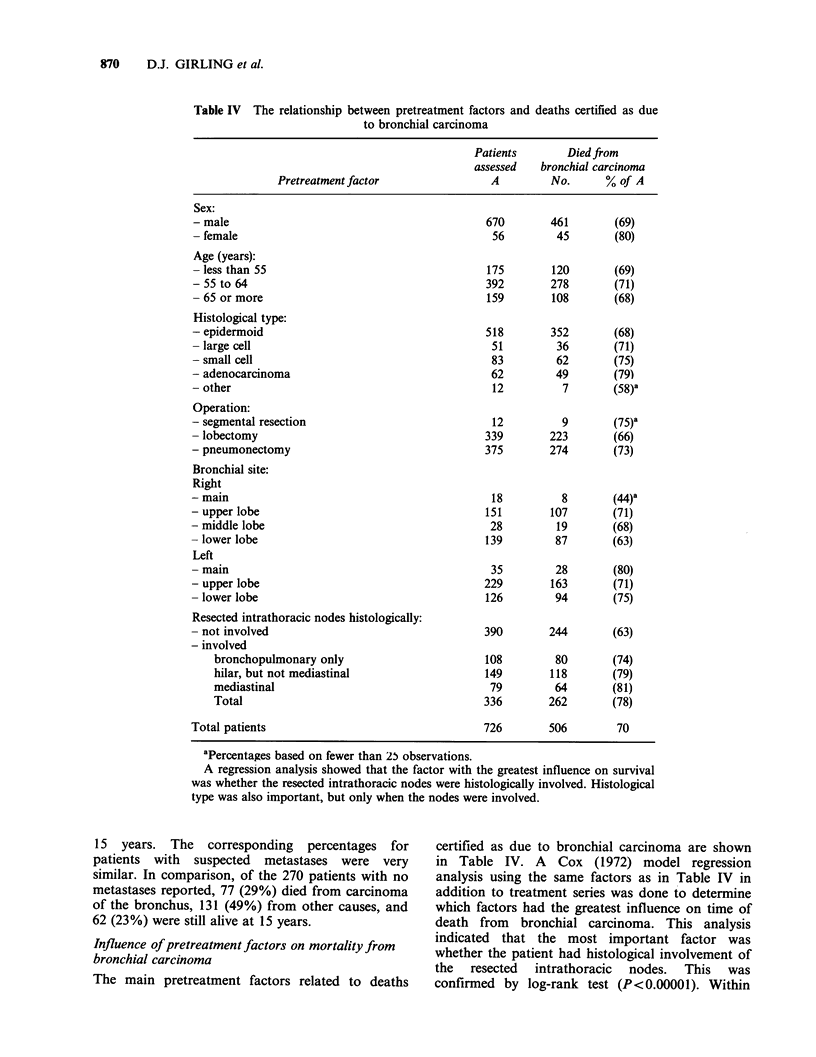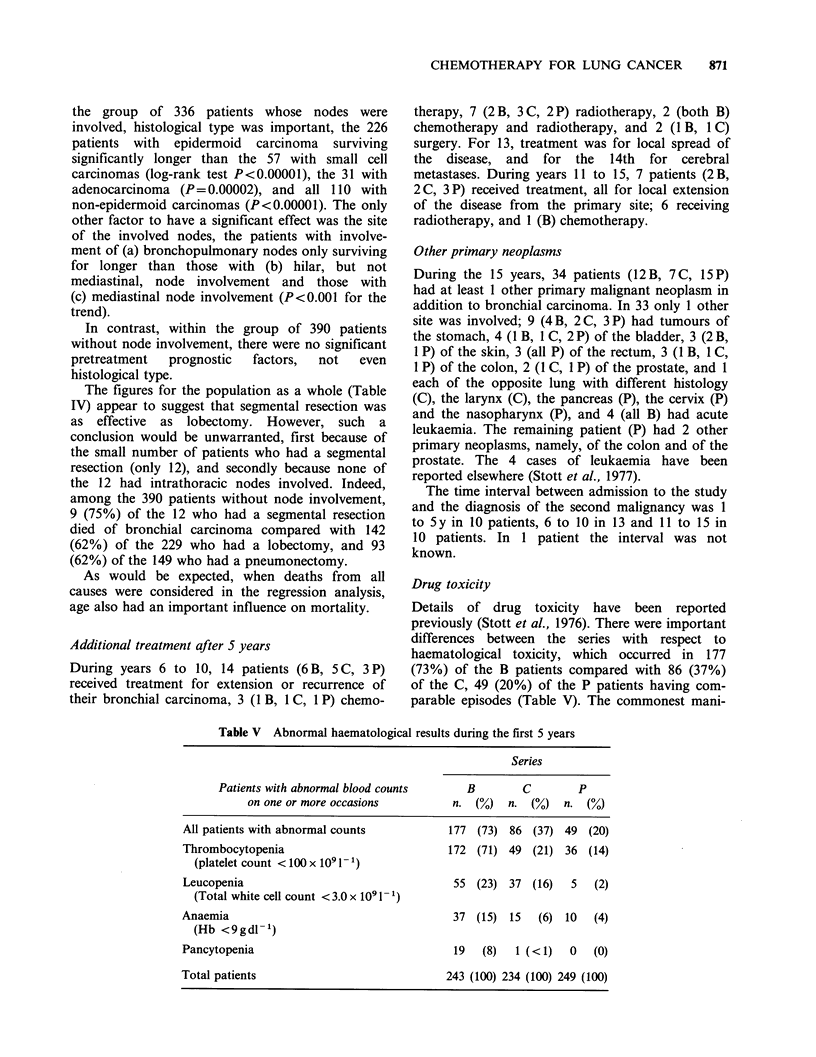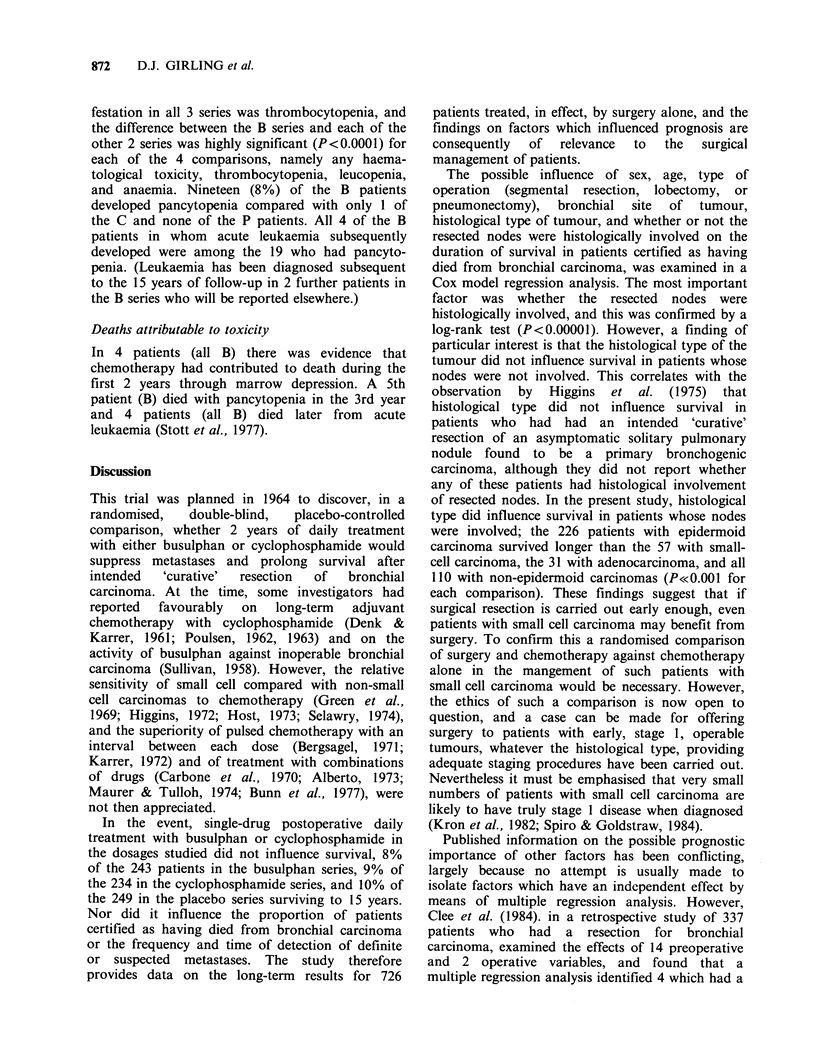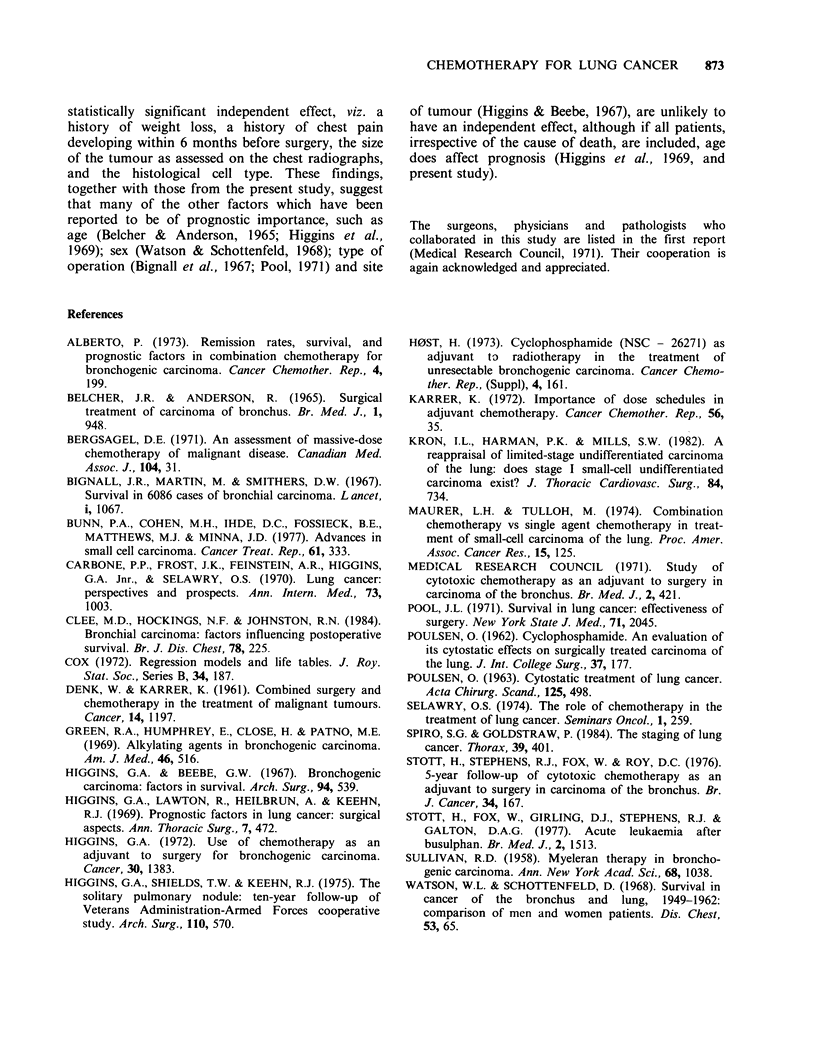# Fifteen-year follow-up of all patients in a study of post-operative chemotherapy for bronchial carcinoma.

**DOI:** 10.1038/bjc.1985.271

**Published:** 1985-12

**Authors:** D. J. Girling, H. Stott, R. J. Stephens, W. Fox

## Abstract

The 15-year findings are presented of a double-blind, randomised study planned in 1964 in which cytotoxic chemotherapy with either busulphan or cyclophosphamide prescribed to be given daily for 2 years as an adjuvant to surgery was compared with placebo in the treatment of 726 patients with carcinoma of the bronchus. The two cytotoxic agents administered in this way did not influence survival. At 15y, 8% of the 243 patients allocated busulphan, 9% of the 234 cyclophosphamide, and 10% of the 249 placebo were alive, these being 10% of the patients who had had epidermoid cancers, 12% large-cell, 5% small-cell, 5% adenocarcinomas, and 8% other histological types. The study provides data on long-term results in a large group of patients who were, in effect, treated by surgery alone. Survival was significantly shorter in patients with histological involvement of the resected intrathoracic nodes (log-rank test P much less than 0.001). A finding of particular interest is that the histological type of the tumour did not influence survival in the 390 patients whose nodes were not involved, although, as expected, it did in the 336 whose nodes were involved, the 226 with epidermoid cancers surviving longer than the 57 with small cell carcinoma, the 31 with adenocarcinoma and all 110 with non-epidermoid carcinomas (P much less than 0.001 in each comparison).


*Br. J. Cancer* (1985), **52**, 867–873

### Fifteen-year follow-up of all patients in a study of post-operative chemotherapy for bronchial carcinoma

D.J. Girling, H. Stott, R.J. Stephens & W. Fox

*Medical Research Council Tuberculosis and Chest Diseases Unit, Brompton Hospital, Fulham Road, London SW3 6HP, UK.*

**Summary** The 15-year findings are presented of a double-blind, randomised study planned in 1964 in which cytotoxic chemotherapy with either busulphan or cyclophosphamide prescribed to be given daily for 2 years as an adjuvant to surgery was compared with placebo in the treatment of 726 patients with carcinoma of the bronchus. The two cytotoxic agents administered in this way did not influence survival. At 15 y, 8% of the 243 patients allocated busulphan, 9% of the 234 cyclophosphamide, and 10% of the 249 placebo were alive, these being 10% of the patients who had had epidermoid cancers, 12% large-cell, 5% small-cell, 5% adenocarcinomas, and 8% other histological types. The study provides data on long-term results in a large group of patients who were, in effect, treated by surgery alone. Survival was significantly shorter in patients with histological involvement of the resected intrathoracic nodes (log-rank test $P \ll 0.001$). A finding of particular interest is that the histological type of the tumour did not influence survival in the 390 patients whose nodes were not involved, although, as expected, it did in the 336 whose nodes were involved, the 226 with epidermoid cancers surviving longer than the 57 with small cell carcinoma, the 31 with adenocarcinoma and all 110 with non-epidermoid carcinomas ($P \ll 0.001$ in each comparison).

The results at 5 years (Stott *et al.*, 1976) of a Medical Research Council Working Party double-blind study, planned in 1964, to find out whether 2 years of daily chemotherapy with busulphan or cyclophosphamide, compared with placebo, following surgical removal of bronchial carcinoma would suppress metastases and prolong survival, showed no benefit from either of these cytotoxic agents in the dosage schedules studied. Moreover, there was a high incidence of haematological toxicity to busulphan. All the survivors have now been studied for 15 years and the findings are presented in this report.

### Plan and conduct of the study

The plan and conduct of the study were described in detail previously (Medical Research Council, 1971). In summary, after resection of all intrathoracic bronchial tumour, the patients were allocated at random to receive tablets of busulphan (B series), cyclophosphamide (C series), or indistinguishable placebos (P series) daily for 2 years. For the first 10 days following operation, all patients received 8 tablets in a single daily dose (B series 4 mg, C series 200 mg). Thereafter they received 6 tablets daily (B series 3 mg, C series 150 mg). However, after about the first year of intake to the study, these maintenance dosages were reduced from 6 to 3 tablets (B series 1.5 mg, C series 75 mg), because of an unexpectedly high incidence of toxicity. The 3 series were very similar with respect to sex, age, type of operation, bronchial site of tumour, histological type of tumour, and whether or not the resected lymph-nodes were histologically involved.

Each patient was reported on monthly during the first 3 years, 3-monthly up to 5 years, and annually thereafter. Total leucocyte and platelet counts and haemoglobin concentrations were measured every month during the first 2 years, and thereafter when requested by the clinician. The maintenance dose of tablets was controlled by the clinician from the results of the blood investigations and the dose was reduced if haematological toxicity was suspected.

The study was conducted double-blind. The certified cause of death was obtained from the local centre and was verified from the death entries at the OPCS for all but 2 of the deaths.

It is important to note that as the study was planned in 1964, some of the peri-operative staging procedures which would now be considered desirable were not required as a routine and when done were not necessarily reported. In particular, the following were not specifically requested as part of the protocol: recording recent weight loss, mediastinoscopy, isotope scans, liver function tests, marrow aspiration or biopsy, routine dissection of the mediastinal nodes at operation, or reporting the size of the tumour in the resected specimen.

Correspondence: D.J. Girling
Received 14 June 1985; and in revised form 21 August, 1985.

© The Macmillan Press Ltd., 1985

### Results

The only important difference between patients prescribed the two maintenance dosage schedules was the incidence of toxicity. The amalgamated results are therefore presented.

### Survival up to 15 years

The survival curves (Figure 1) show no statistically significant difference between the 3 series (log-rank tests). Even within the group of 83 patients with small cell carcinoma, treatment series had no effect on survival. The survivors at 5, 10 and 15 y are shown in Table I according to the series and the histological type of carcinoma. At 15 y, 8% of the B, 9% of the C and 10% of the P patients were alive, these being 10% of the patients with epidermoid cancers, 12% of those with large cell cancers, 5% of those with small cell cancers, 5% of

**Figure 1** Survival from date of allocation.

**Table I** Survivors at 5, 10, and 15 years according to series and histological type of tumour

| | Total patients | *Survivors at* 5 years n. (%) | *Survivors at* 10 years n. (%) | *Survivors at* 15 years n. (%) |
|---|---|---|---|---|
| Series: | | | | |
| Busulphan | 243 | 69 (28) | 34 (14) | 20 (8) |
| Cyclophosphamide | 234 | 63 (27) | 33 (14) | 21 (9) |
| Placebo | 249 | 85 (34) | 59 (24) | 25 (10) |
| Histological type: | | | | |
| – epidermoid | 518 | 176 (34) | 99 (19) | 52 (10) |
| – large cell | 51 | 12 (24) | 10 (20) | 6 (12) |
| – small cell | 83 | 12 (14) | 7 (8) | 4 (5) |
| – adenocarcinoma | 62 | 11 (18) | 5 (8) | 3 (5) |
| – other | 12 | 6 (50)[a] | 5 (42)[a] | 1 (8)[a] |
| Total patients | 726 | 217 (30) | 126 (17) | 66 (9) |

[a]Percentages based on fewer than 25 observations.

those with adenocarcinomas, and 8% of those with other histological types.

The mean age of the 66 survivors at 15 y was 71 y (range 49–85). Their general condition was reported as 'good' for 49 (74%), 'fair' for 15 (23%), and 'poor' for only 2 (3%); 38 (58%) were fully active, a further 25 (38%) were out and about but with restricted activity, and only 3 (5%) were confined to home or hospital or were bedridden.

### *Cause of death*

The proportions of patients certified as having died from bronchial carcinoma were similar in the 3 series. By 5 y, 144 (59%) of the B, 142 (61%) of the C, and 141 (57%) of the P patients had been so certified. By 10 y, these figures had risen to 167 (69%), 160 (68%), and 156 (63%), and by 15 y to 171 (70%), 167 (71%), and 168 (67%), respectively. The proportion of total deaths due to bronchial carcinoma was 427 (84%) of the 509 during the first 5 years, compared with 56 (62%) of the 91 during years 6 to 10, and 23 (38%) of the 60 during years 11 to 15, there being no statistically significant differences between the 3 series.

### *Evidence of metastases*

Metastases were reported to have been definitely present at some time during the 15 y (Table II) in 131 (54%) of the 243 B, 111 (47%) of the 234 C, and 132 (53%) of the 249 P patients, and had been suspected in a further 25 (10%), 29 (12%), and 28 (11%) respectively. Metastases appeared after similar periods in the 3 series, most of them during the first 5 y, namely in 340 (91%) of the 374 patients with definite, and in 77 (94%) of the 82 with suspected metastases.

Of the 374 patients (Table III) with definite metastases reported, 352 (94%) died from carcinoma of the bronchus, 18 (5%) from other causes, and the remaining 4 (1%) were still alive at

**Table II** Cumulative totals of patients who had had definite and suspected metastases reported by 5, 10, and 15 years

| | | | *Patients with metastases by:* | | |
|---|---|---|---|---|---|
| *Series* | *Total patients* | *Metastases* | *5 years n. (%)* | *10 years n. (%)* | *15 years n. (%)* |
| Busulphan | 243 | definite | 118 (49) | 129 (53) | 131 (54) |
| | | suspected | 23 (9) | 24 (10) | 25 (10) |
| | | none | 102 (42) | 90 (37) | 87 (36) |
| Cyclophosphamide | 234 | definite | 102 (44) | 109 (47) | 111 (47) |
| | | suspected | 29 (12) | 29 (12) | 29 (12) |
| | | none | 103 (44) | 96 (41) | 94 (40) |
| Placebo | 249 | definite | 120 (48) | 125 (50) | 132 (53) |
| | | suspected | 25 (10) | 26 (10) | 28 (11) |
| | | none | 104 (42) | 98 (39) | 89 (36) |
| All series | 726 | definite | 340 (47) | 363 (50) | 374 (52) |
| | | suspected | 77 (11) | 79 (11) | 82 (11) |
| | | none | 309 (43) | 284 (39) | 270 (37) |

**Table III** The relationship between the presence of metastases and the outcome at 15 years

| | *Evidence of metastases by 15 years* | | | |
|---|---|---|---|---|
| *Outcome at 15 years* | *Definite n. (%)* | *Suspected n. (%)* | *None n. (%)* | *Total n. (%)* |
| Died from carcinoma of the bronchus | 352 (94) | 77 (94) | 77 (29) | 506 (70) |
| Died from other causes | 18 (5) | 5 (6) | 131 (49) | 154 (21) |
| Alive | 4 (1) | 0 (0) | 62 (23) | 66 (9) |
| Total | 374 (100) | 82 (100) | 270 (100) | 726 (100) |

**Table IV** The relationship between pretreatment factors and deaths certified as due to bronchial carcinoma

| *Pretreatment factor* | *Patients assessed A* | *Died from bronchial carcinoma No.* | *% of A* |
|---|---|---|---|
| Sex: | | | |
| – male | 670 | 461 | (69) |
| – female | 56 | 45 | (80) |
| Age (years): | | | |
| – less than 55 | 175 | 120 | (69) |
| – 55 to 64 | 392 | 278 | (71) |
| – 65 or more | 159 | 108 | (68) |
| Histological type: | | | |
| – epidermoid | 518 | 352 | (68) |
| – large cell | 51 | 36 | (71) |
| – small cell | 83 | 62 | (75) |
| – adenocarcinoma | 62 | 49 | (79) |
| – other | 12 | 7 | (58)[a] |
| Operation: | | | |
| – segmental resection | 12 | 9 | (75)[a] |
| – lobectomy | 339 | 223 | (66) |
| – pneumonectomy | 375 | 274 | (73) |
| Bronchial site: | | | |
| Right | | | |
| – main | 18 | 8 | (44)[a] |
| – upper lobe | 151 | 107 | (71) |
| – middle lobe | 28 | 19 | (68) |
| – lower lobe | 139 | 87 | (63) |
| Left | | | |
| – main | 35 | 28 | (80) |
| – upper lobe | 229 | 163 | (71) |
| – lower lobe | 126 | 94 | (75) |
| Resected intrathoracic nodes histologically: | | | |
| – not involved | 390 | 244 | (63) |
| – involved | | | |
| bronchopulmonary only | 108 | 80 | (74) |
| hilar, but not mediastinal | 149 | 118 | (79) |
| mediastinal | 79 | 64 | (81) |
| Total | 336 | 262 | (78) |
| Total patients | 726 | 506 | 70 |

[a]Percentages based on fewer than 25 observations.

A regression analysis showed that the factor with the greatest influence on survival was whether the resected intrathoracic nodes were histologically involved. Histological type was also important, but only when the nodes were involved.

15 years. The corresponding percentages for patients with suspected metastases were very similar. In comparison, of the 270 patients with no metastases reported, 77 (29%) died from carcinoma of the bronchus, 131 (49%) from other causes, and 62 (23%) were still alive at 15 years.

*Influence of pretreatment factors on mortality from bronchial carcinoma*

The main pretreatment factors related to deaths certified as due to bronchial carcinoma are shown in Table IV. A Cox (1972) model regression analysis using the same factors as in Table IV in addition to treatment series was done to determine which factors had the greatest influence on time of death from bronchial carcinoma. This analysis indicated that the most important factor was whether the patient had histological involvement of the resected intrathoracic nodes. This was confirmed by log-rank test ($P<0.00001$). Within the group of 336 patients whose nodes were involved, histological type was important, the 226 patients with epidermoid carcinoma surviving significantly longer than the 57 with small cell carcinomas (log-rank test $P<0.00001$), the 31 with adenocarcinoma ($P=0.00002$), and all 110 with non-epidermoid carcinomas ($P<0.00001$). The only other factor to have a significant effect was the site of the involved nodes, the patients with involvement of (a) bronchopulmonary nodes only surviving for longer than those with (b) hilar, but not mediastinal, node involvement and those with (c) mediastinal node involvement ($P<0.001$ for the trend).

In contrast, within the group of 390 patients without node involvement, there were no significant pretreatment prognostic factors, not even histological type.

The figures for the population as a whole (Table IV) appear to suggest that segmental resection was as effective as lobectomy. However, such a conclusion would be unwarranted, first because of the small number of patients who had a segmental resection (only 12), and secondly because none of the 12 had intrathoracic nodes involved. Indeed, among the 390 patients without node involvement, 9 (75%) of the 12 who had a segmental resection died of bronchial carcinoma compared with 142 (62%) of the 229 who had a lobectomy, and 93 (62%) of the 149 who had a pneumonectomy.

As would be expected, when deaths from all causes were considered in the regression analysis, age also had an important influence on mortality.

### *Additional treatment after 5 years*

During years 6 to 10, 14 patients (6 B, 5 C, 3 P) received treatment for extension or recurrence of their bronchial carcinoma, 3 (1 B, 1 C, 1 P) chemotherapy, 7 (2 B, 3 C, 2 P) radiotherapy, 2 (both B) chemotherapy and radiotherapy, and 2 (1 B, 1 C) surgery. For 13, treatment was for local spread of the disease, and for the 14th for cerebral metastases. During years 11 to 15, 7 patients (2 B, 2 C, 3 P) received treatment, all for local extension of the disease from the primary site; 6 receiving radiotherapy, and 1 (B) chemotherapy.

### *Other primary neoplasms*

During the 15 years, 34 patients (12 B, 7 C, 15 P) had at least 1 other primary malignant neoplasm in addition to bronchial carcinoma. In 33 only 1 other site was involved; 9 (4 B, 2 C, 3 P) had tumours of the stomach, 4 (1 B, 1 C, 2 P) of the bladder, 3 (2 B, 1 P) of the skin, 3 (all P) of the rectum, 3 (1 B, 1 C, 1 P) of the colon, 2 (1 C, 1 P) of the prostate, and 1 each of the opposite lung with different histology (C), the larynx (C), the pancreas (P), the cervix (P) and the nasopharynx (P), and 4 (all B) had acute leukaemia. The remaining patient (P) had 2 other primary neoplasms, namely, of the colon and of the prostate. The 4 cases of leukaemia have been reported elsewhere (Stott *et al.*, 1977).

The time interval between admission to the study and the diagnosis of the second malignancy was 1 to 5 y in 10 patients, 6 to 10 in 13 and 11 to 15 in 10 patients. In 1 patient the interval was not known.

### *Drug toxicity*

Details of drug toxicity have been reported previously (Stott *et al.*, 1976). There were important differences between the series with respect to haematological toxicity, which occurred in 177 (73%) of the B patients compared with 86 (37%) of the C, 49 (20%) of the P patients having comparable episodes (Table V). The commonest mani-

**Table V** Abnormal haematological results during the first 5 years

| | *Series* | | | | | |
|---|---|---|---|---|---|---|
| *Patients with abnormal blood counts on one or more occasions* | *B* *n.* | (%) | *C* *n.* | (%) | *P* *n.* | (%) |
| All patients with abnormal counts | 177 | (73) | 86 | (37) | 49 | (20) |
| Thrombocytopenia (platelet count $<100\times10^9\,l^{-1}$) | 172 | (71) | 49 | (21) | 36 | (14) |
| Leucopenia (Total white cell count $<3.0\times10^9\,l^{-1}$) | 55 | (23) | 37 | (16) | 5 | (2) |
| Anaemia (Hb $<9\,g\,dl^{-1}$) | 37 | (15) | 15 | (6) | 10 | (4) |
| Pancytopenia | 19 | (8) | 1 | (<1) | 0 | (0) |
| Total patients | 243 | (100) | 234 | (100) | 249 | (100) |

festation in all 3 series was thrombocytopenia, and the difference between the B series and each of the other 2 series was highly significant ($P < 0.0001$) for each of the 4 comparisons, namely any haematological toxicity, thrombocytopenia, leucopenia, and anaemia. Nineteen (8%) of the B patients developed pancytopenia compared with only 1 of the C and none of the P patients. All 4 of the B patients in whom acute leukaemia subsequently developed were among the 19 who had pancytopenia. (Leukaemia has been diagnosed subsequent to the 15 years of follow-up in 2 further patients in the B series who will be reported elsewhere.)

*Deaths attributable to toxicity*

In 4 patients (all B) there was evidence that chemotherapy had contributed to death during the first 2 years through marrow depression. A 5th patient (B) died with pancytopenia in the 3rd year and 4 patients (all B) died later from acute leukaemia (Stott *et al.*, 1977).

### Discussion

This trial was planned in 1964 to discover, in a randomised, double-blind, placebo-controlled comparison, whether 2 years of daily treatment with either busulphan or cyclophosphamide would suppress metastases and prolong survival after intended 'curative' resection of bronchial carcinoma. At the time, some investigators had reported favourably on long-term adjuvant chemotherapy with cyclophosphamide (Denk & Karrer, 1961; Poulsen, 1962, 1963) and on the activity of busulphan against inoperable bronchial carcinoma (Sullivan, 1958). However, the relative sensitivity of small cell compared with non-small cell carcinomas to chemotherapy (Green *et al.*, 1969; Higgins, 1972; Host, 1973; Selawry, 1974), and the superiority of pulsed chemotherapy with an interval between each dose (Bergsagel, 1971; Karrer, 1972) and of treatment with combinations of drugs (Carbone *et al.*, 1970; Alberto, 1973; Maurer & Tulloh, 1974; Bunn *et al.*, 1977), were not then appreciated.

In the event, single-drug postoperative daily treatment with busulphan or cyclophosphamide in the dosages studied did not influence survival, 8% of the 243 patients in the busulphan series, 9% of the 234 in the cyclophosphamide series, and 10% of the 249 in the placebo series surviving to 15 years. Nor did it influence the proportion of patients certified as having died from bronchial carcinoma or the frequency and time of detection of definite or suspected metastases. The study therefore provides data on the long-term results for 726 patients treated, in effect, by surgery alone, and the findings on factors which influenced prognosis are consequently of relevance to the surgical management of patients.

The possible influence of sex, age, type of operation (segmental resection, lobectomy, or pneumonectomy), bronchial site of tumour, histological type of tumour, and whether or not the resected nodes were histologically involved on the duration of survival in patients certified as having died from bronchial carcinoma, was examined in a Cox model regression analysis. The most important factor was whether the resected nodes were histologically involved, and this was confirmed by a log-rank test ($P < 0.00001$). However, a finding of particular interest is that the histological type of the tumour did not influence survival in patients whose nodes were not involved. This correlates with the observation by Higgins *et al.* (1975) that histological type did not influence survival in patients who had had an intended 'curative' resection of an asymptomatic solitary pulmonary nodule found to be a primary bronchogenic carcinoma, although they did not report whether any of these patients had histological involvement of resected nodes. In the present study, histological type did influence survival in patients whose nodes were involved; the 226 patients with epidermoid carcinoma survived longer than the 57 with small-cell carcinoma, the 31 with adenocarcinoma, and all 110 with non-epidermoid carcinomas ($P \ll 0.001$ for each comparison). These findings suggest that if surgical resection is carried out early enough, even patients with small cell carcinoma may benefit from surgery. To confirm this a randomised comparison of surgery and chemotherapy against chemotherapy alone in the mangement of such patients with small cell carcinoma would be necessary. However, the ethics of such a comparison is now open to question, and a case can be made for offering surgery to patients with early, stage 1, operable tumours, whatever the histological type, providing adequate staging procedures have been carried out. Nevertheless it must be emphasised that very small numbers of patients with small cell carcinoma are likely to have truly stage 1 disease when diagnosed (Kron *et al.*, 1982; Spiro & Goldstraw, 1984).

Published information on the possible prognostic importance of other factors has been conflicting, largely because no attempt is usually made to isolate factors which have an independent effect by means of multiple regression analysis. However, Clee *et al.* (1984). in a retrospective study of 337 patients who had a resection for bronchial carcinoma, examined the effects of 14 preoperative and 2 operative variables, and found that a multiple regression analysis identified 4 which had a statistically significant independent effect, *viz.* a history of weight loss, a history of chest pain developing within 6 months before surgery, the size of the tumour as assessed on the chest radiographs, and the histological cell type. These findings, together with those from the present study, suggest that many of the other factors which have been reported to be of prognostic importance, such as age (Belcher & Anderson, 1965; Higgins *et al.*, 1969); sex (Watson & Schottenfeld, 1968); type of operation (Bignall *et al.*, 1967; Pool, 1971) and site of tumour (Higgins & Beebe, 1967), are unlikely to have an independent effect, although if all patients, irrespective of the cause of death, are included, age does affect prognosis (Higgins *et al.*, 1969, and present study).

The surgeons, physicians and pathologists who collaborated in this study are listed in the first report (Medical Research Council, 1971). Their cooperation is again acknowledged and appreciated.

**References**

ALBERTO, P. (1973). Remission rates, survival, and prognostic factors in combination chemotherapy for bronchogenic carcinoma. *Cancer Chemother. Rep.*, **4**, 199.

BELCHER, J.R. & ANDERSON, R. (1965). Surgical treatment of carcinoma of bronchus. *Br. Med. J.*, **1**, 948.

BERGSAGEL, D.E. (1971). An assessment of massive-dose chemotherapy of malignant disease. *Canadian Med. Assoc. J.*, **104**, 31.

BIGNALL, J.R., MARTIN, M. & SMITHERS, D.W. (1967). Survival in 6086 cases of bronchial carcinoma. *Lancet*, **i**, 1067.

BUNN, P.A., COHEN, M.H., IHDE, D.C., FOSSIECK, B.E., MATTHEWS, M.J. & MINNA, J.D. (1977). Advances in small cell carcinoma. *Cancer Treat. Rep.*, **61**, 333.

CARBONE, P.P., FROST, J.K., FEINSTEIN, A.R., HIGGINS, G.A. Jnr., & SELAWRY, O.S. (1970). Lung cancer: perspectives and prospects. *Ann. Intern. Med.*, **73**, 1003.

CLEE, M.D., HOCKINGS, N.F. & JOHNSTON, R.N. (1984). Bronchial carcinoma: factors influencing postoperative survival. *Br. J. Dis. Chest*, **78**, 225.

COX (1972). Regression models and life tables. *J. Roy. Stat. Soc.*, Series B, **34**, 187.

DENK, W. & KARRER, K. (1961). Combined surgery and chemotherapy in the treatment of malignant tumours. *Cancer*, **14**, 1197.

GREEN, R.A., HUMPHREY, E., CLOSE, H. & PATNO, M.E. (1969). Alkylating agents in bronchogenic carcinoma. *Am. J. Med.*, **46**, 516.

HIGGINS, G.A. & BEEBE, G.W. (1967). Bronchogenic carcinoma: factors in survival. *Arch. Surg.*, **94**, 539.

HIGGINS, G.A., LAWTON, R., HEILBRUN, A. & KEEHN, R.J. (1969). Prognostic factors in lung cancer: surgical aspects. *Ann. Thoracic Surg.*, **7**, 472.

HIGGINS, G.A. (1972). Use of chemotherapy as an adjuvant to surgery for bronchogenic carcinoma. *Cancer*, **30**, 1383.

HIGGINS, G.A., SHIELDS, T.W. & KEEHN, R.J. (1975). The solitary pulmonary nodule: ten-year follow-up of Veterans Administration-Armed Forces cooperative study. *Arch. Surg.*, **110**, 570.

HØST, H. (1973). Cyclophosphamide (NSC – 26271) as adjuvant to radiotherapy in the treatment of unresectable bronchogenic carcinoma. *Cancer Chemother. Rep.*, (Suppl), **4**, 161.

KARRER, K. (1972). Importance of dose schedules in adjuvant chemotherapy. *Cancer Chemother. Rep.*, **56**, 35.

KRON, I.L., HARMAN, P.K. & MILLS, S.W. (1982). A reappraisal of limited-stage undifferentiated carcinoma of the lung: does stage I small-cell undifferentiated carcinoma exist? *J. Thoracic Cardiovasc. Surg.*, **84**, 734.

MAURER, L.H. & TULLOH, M. (1974). Combination chemotherapy vs single agent chemotherapy in treatment of small-cell carcinoma of the lung. *Proc. Amer. Assoc. Cancer Res.*, **15**, 125.

MEDICAL RESEARCH COUNCIL (1971). Study of cytotoxic chemotherapy as an adjuvant to surgery in carcinoma of the bronchus. *Br. Med. J.*, **2**, 421.

POOL, J.L. (1971). Survival in lung cancer: effectiveness of surgery. *New York State J. Med.*, **71**, 2045.

POULSEN, O. (1962). Cyclophosphamide. An evaluation of its cytostatic effects on surgically treated carcinoma of the lung. *J. Int. College Surg.*, **37**, 177.

POULSEN, O. (1963). Cytostatic treatment of lung cancer. *Acta Chirurg. Scand.*, **125**, 498.

SELAWRY, O.S. (1974). The role of chemotherapy in the treatment of lung cancer. *Seminars Oncol.*, **1**, 259.

SPIRO, S.G. & GOLDSTRAW, P. (1984). The staging of lung cancer. *Thorax*, **39**, 401.

STOTT, H., STEPHENS, R.J., FOX, W. & ROY, D.C. (1976). 5-year follow-up of cytotoxic chemotherapy as an adjuvant to surgery in carcinoma of the bronchus. *Br. J. Cancer*, **34**, 167.

STOTT, H., FOX, W., GIRLING, D.J., STEPHENS, R.J. & GALTON, D.A.G. (1977). Acute leukaemia after busulphan. *Br. Med. J.*, **2**, 1513.

SULLIVAN, R.D. (1958). Myeleran therapy in bronchogenic carcinoma. *Ann. New York Acad. Sci.*, **68**, 1038.

WATSON, W.L. & SCHOTTENFELD, D. (1968). Survival in cancer of the bronchus and lung, 1949–1962: comparison of men and women patients. *Dis. Chest*, **53**, 65.